# Effects of early life exposure to ultraviolet C radiation on mitochondrial DNA content, transcription, ATP production, and oxygen consumption in developing *Caenorhabditis elegans*

**DOI:** 10.1186/2050-6511-14-9

**Published:** 2013-02-04

**Authors:** Maxwell CK Leung, John P Rooney, Ian T Ryde, Autumn J Bernal, Amanda S Bess, Tracey L Crocker, Alex Q Ji, Joel N Meyer

**Affiliations:** 1 Nicholas School of the Environment, Duke University, Durham, NC, USA; 2 Integrated Toxicology and Environmental Health Program, Duke University, Durham, NC, USA

**Keywords:** *Caenorhabditis elegans*, Mitochondrial DNA damage, Mitochondrial dysfunction, Ultraviolet C radiation, Early life exposure, Genotoxicity

## Abstract

**Background:**

Mitochondrial DNA (mtDNA) is present in multiple copies per cell and undergoes dramatic amplification during development. The impacts of mtDNA damage incurred early in development are not well understood, especially in the case of types of mtDNA damage that are irreparable, such as ultraviolet C radiation (UVC)-induced photodimers.

**Methods:**

We exposed first larval stage nematodes to UVC using a protocol that results in accumulated mtDNA damage but permits nuclear DNA (nDNA) repair. We then measured the transcriptional response, as well as oxygen consumption, ATP levels, and mtDNA copy number through adulthood.

**Results:**

Although the mtDNA damage persisted to the fourth larval stage, we observed only a relatively minor ~40% decrease in mtDNA copy number. Transcriptomic analysis suggested an inhibition of aerobic metabolism and developmental processes; mRNA levels for mtDNA-encoded genes were reduced ~50% at 3 hours post-treatment, but recovered and, in some cases, were upregulated at 24 and 48 hours post-exposure. The mtDNA polymerase γ was also induced ~8-fold at 48 hours post-exposure. Moreover, ATP levels and oxygen consumption were reduced in response to UVC exposure, with marked reductions of ~50% at the later larval stages.

**Conclusions:**

These results support the hypothesis that early life exposure to mitochondrial genotoxicants could result in mitochondrial dysfunction at later stages of life, thereby highlighting the potential health hazards of time-delayed effects of these genotoxicants in the environment.

## Background

In recent years, potential environmental effects on mitochondrial biology have attracted increasing research interest [1,2]. Mitochondrial DNA (mtDNA) is more sensitive than nuclear DNA (nDNA) to exposure to some chemicals, perhaps due to the absence of chromatin packing and many DNA repair pathways in mitochondria [3]. The high lipid content of the mitochondrial membranes and the slightly negative charge of the mitochondrial matrix also attract lipophilic or positively charged compounds to mitochondria [4]. Furthermore, non-genotoxic mitochondrial toxicants might disrupt mitochondrial function and indirectly cause mtDNA damage via generation of reactive oxygen species [5].

Theoretical considerations and some empirical data suggest that mtDNA damage that occurs at different stages of human life may lead to very different physiological effects. Since the quality of mitochondria in differentiated tissues depends on the quality of mitochondria in their precursors, mitochondrial damage in the early stages of human development may potentially affect mature tissue function. For example, mitochondrial toxicities exerted by developmental exposure to anti-HIV drugs in humans and laboratory models [6] demonstrate the importance of normal mtDNA biology during development.

The mitochondrial biology of *Caenorhabditis elegans* is generally similar to that of humans [7]. The genome is 13,794 base pairs in length (Additional file 1: Figure S1), compared to 16,649 in humans. The genes encoded appear to be identical; while an *atp-8* gene has not been definitively identified in *C. elegans*, it is probably present with an unusual sequence, as is the case in other nematodes [8]. There are also indications that the developmental biology of mitochondria is similar in *C. elegans* and humans: *C. elegans*[7,9,10], like humans [11,12], shows a large increase in mtDNA copy number with age as well as a switch from anaerobic to aerobic metabolism during development. Thus, *C. elegans* offers a useful model for the *in vivo* study of mitochondrial biology, as well as the response to toxicants [13].

Recent work by Furda et al. [14] demonstrated that persistent mtDNA damage can lead to mitochondrial dysfunction, but the response was dependent on the type of DNA damage incurred. We recently described a serial ultraviolet C radiation (UVC) exposure protocol that resulted in a large amount of irreparable mtDNA damage in *C. elegans*, but permitted the repair of the nDNA damage [15]. UVC creates photodimers almost exclusively, and previous *in vitro* evidence [16,17] suggests that such damage might inhibit mtDNA replication and transcription *in vivo*. In this work, we investigated the hypothesis that early life exposure to serial UVC results in later life mitochondrial dysfunction.

## Methods

### *C. elegans* culture and exposures

*C. elegans* were cultured and exposed to UVC during the L1 stage, largely as previously described [15]. Briefly, synchronized L1 larvae were produced by overnight hatch in M9 medium following bleach-sodium hydroxide isolation of eggs as previously described [18]. The L1 larvae were placed on peptone-free (to prevent inadvertent microbial growth) K agar plates with or without 5 μg/mL ethidium bromide (EtBr) for 48 h without food at 20°C. Half of the plates were also exposed to 7.5 J/m^2^ UVC radiation at 0, 24, and 48 h as described [15], and then transferred to OP50-seeded plates. The UVC exposure protocol is based on the fact that UVC-induced DNA damage is quickly repaired in the nuclear but not mitochondrial genome [15,19], thus allowing for accumulation of mitochondrial DNA damage while permitting repair of nuclear DNA. This protocol results in no larval growth delay prior to the L4 stage [15]. The transgenic strain PE255 expressing firefly luciferase as an *in vivo* reporter for ATP level [20] was generously provided by Dr. Cristina Lagido (University of Aberdeen, UK). The wildtype strain N2 was obtained from *Caenorhabditis* Genetics Center (University of Minnesota), which is funded by the NIH National Center for Research Resources (NCRR).

### Microarray experiments

**Figure 1:**
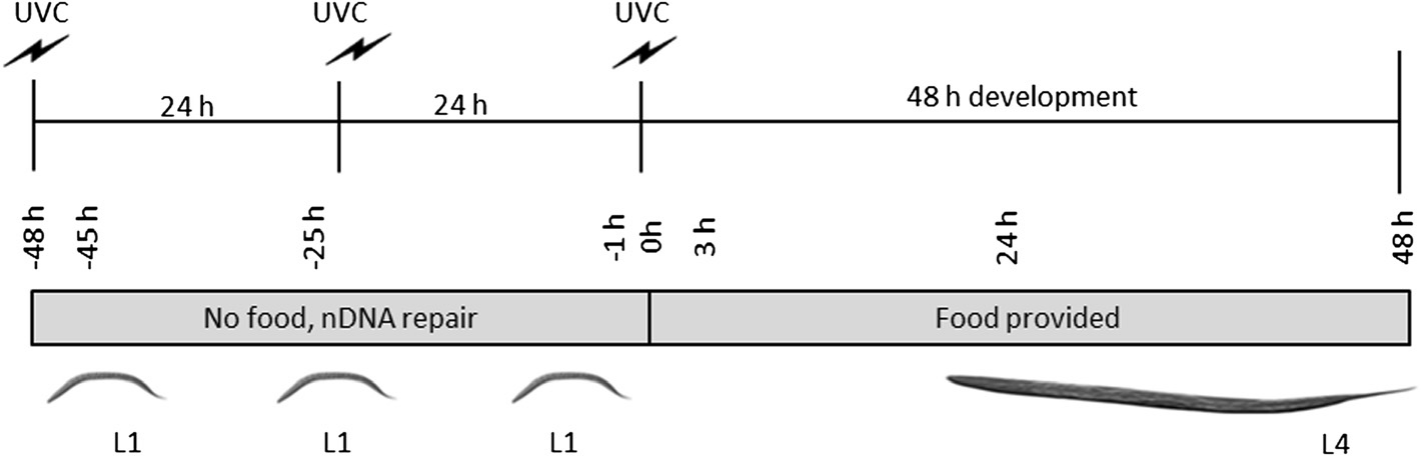
**Experimental design.** Liquid-hatched L1 stage C. elegans were exposed to 7.5 J/m^2^ UVC over 48 h, in the absence of food, permitting nDNA repair but accumulation of mtDNA damage [15]. Nematodes were then placed on food plates and followed for another 48 h. We measured mRNA levels, genome copy number, DNA damage, ATP levels, and oxygen consumption at multiple times during and after the UVC exposures. All times are given relative to the final dose and transferral to food (= “0 h”). For example, mRNA sampled immediately prior to the second dose of UVC would be described as sampled at “-25 h”, and “3 h” if it were sampled 3 h after the final UVC exposure. Representative examples are presented to orient the reader.

N2 nematodes were sampled for RNA isolation at 3 h after the first UVC exposure, 1 h prior to the second exposure, 1 h prior to the third exposure, and 3 h after the third exposure, and transferred to OP50 plates (schematic presented in Figure 1). Transfer to OP50 plates and isolation for freezing were accomplished by washing nematodes off of plates into a 15 mL tube with K-medium, pelleting by centrifugation, followed by two additional cycles of resuspension and centrifugation as described [19]. Nematodes were frozen by dripping 3,000-5,000 pelleted nematodes suspended in about 500 μl K-medium into liquid nitrogen, and stored at −80°C. The pellets were ground into fine powder with a liquid nitrogen-cooled mortar and pestle and RNA was extracted using an RNeasy kit (Qiagen, Valencia, CA, USA). RNA was quantified with a NanoDrop 8000 spectrophotometer (Thermo Scientific/NanoDrop, Wilmington, DE, USA) and analyzed for integrity with an Agilent 2100 BioAnalyzer G2939A (Agilent Technologies, Santa Clara, CA, USA). These exposures were carried out seven times. The seven replicates generated a total “n” of between 4 and 6 for each treatment and timepoint, after excluding samples lost due to insufficient mRNA quality or principle components analysis-based identification of outliers.

Gene expression analysis was conducted using Affymetrix /*C. elegans*/ GeneChip® arrays (Affymetrix, Santa Clara, CA). Hybridization targets were prepared with MessageAmp™ Premier RNA Amplification Kit (Applied Biosystems/Ambion, Austin, TX) from total RNA, hybridized to GeneChip® *C. elegans* Genome Arrays in Affymetrix GeneChip® hybridization oven 645, washed in Affymetrix GeneChip® Fluidics Station 450 and scanned with Affymetrix GeneChip® Scanner 7 G according to standard Affymetrix GeneChip® Hybridization, Wash, and Stain protocols (Affymetrix, Santa Clara,CA). Microarray data have been deposited in the National Center for Biotechnology Information’s GEO and are accessible through GEO series accession number GSE38997.

### Microarray data preprocessing, normalization, error modeling, and initial visualization

We used Principal Components Analysis (PCA) followed by pairwise correlation analysis on unfiltered data to identify outlier samples. Data preprocessing, normalization, and error modeling were performed with Rosetta Resolver® after grouping biological replicates. The resulting fold-changes and p-values were used for Cytoscape analyses (described below).

### GeneSpring-based analysis of microarray data

We used GeneSpring version GX11 (Agilent) to carry out ANOVA, PCA analysis, gene ontology (GO) enrichment analysis, and some visualization.

### Interactome-based analysis of microarray data

Cytoscape (version 2.8.2) was used to overlay microarray data onto two interactomes: the high-confidence WI8 interactome compiled by the Vidal lab [21], and an interactome built by joining four previously-described interactomes: the integrated_function_network from the Vidal lab [21]; an interactome that we assembled previously [19] based on the BIND database [22] and Zhong and Sternberg [23] interactome; the “core” interactome described by Lee et al. [24]; and the higher-probability interactome generated by Alexeyenko and Sonnhammer [25] using a probability of functional coupling cut-off of 0.75. These networks are referred to herein as “WI8” and “Union4” and are composed of 2500 nodes and 3706 edges, and 14334 nodes and 346484 edges, respectively. The Union4 interactome is presented as Additional file 2 in .sif format. jActiveModules (version 2.23, [26]) was used to find, via greedy searching, the top 10 modules with a maximum overlap of 0.3, as identified at a search depth of 1 and maximum depth from start nodes of 2 (with the WI8 interactome) or 1 (with the Union4 interactome). The resulting subnetworks were analyzed for Gene Ontology enrichment with the BiNGO (version 2.44) plugin [27]. We assessed overrepresentation using the hypergeometric test with the Benjamini and Hochberg False Discovery Rate multiple testing correction and significance level of 0.05, testing each cluster versus the entire annotation and identifying altered GO Biological Processes.

### mtDNA and nDNA copy number measurements

mtDNA copy number was measured in N2 and PE255 nematodes using a modification of the real-time PCR assay described by Bratic *et al.*[28]; this assay is based on a plasmid DNA-based standard curve and so generates actual rather than relative copy numbers. The only change to the assay was the use of 2-fold rather than 10-fold dilutions in the standard curve. nDNA copy number was measured using primers designed with Primer 3 [29]: forward - 5^′^-GCC GAC TGG AAG AAC TTG TC-3^′^; reverse - 5^′^-GCG GAG ATC ACC TTC CAG TA-3^′^. These primers amplify a 164 bp region of the gene W09C5.8 (*cox-4*). Nuclear copy number was determined by creating a standard curve for the nuclear DNA based on young adult (24 h post-L4) *glp-1* mutant nematodes raised at 25°C. At this temperature, this strain has a fixed number of cells since it has no germline proliferation [30] and *C. elegans* somatic cells do not divide in adulthood [31]. We based the standard curve on the calculation that adults lacking germ cell proliferation would contain 3134 genomic copies [32,33]. Real-time PCR was carried out in a 7300 Real Time PCR System (Applied Biosystems), under the following conditions: 2 min at 50°C, 10 min at 95°C, 40 cycles of 15 sec at 95°C and 60 sec at 60°C. A dissociation curve was calculated for each sample at the end of each profile. The 25 μl PCR reactions contained 12.5 μl of SYBR Green PCR Master Mix (Applied Biosystems), 8.5 μl H_2_0, 2 μl of target-specific primers at 400 nM final concentration, and 2 μl of nematode lysate obtained as described [34]. The ABI PRISM 7300 Sequence Detection System Software, Version 1.1 (Applied Biosystems) was used to carry out data analysis. All samples were run in triplicate and triplicates were averaged prior to analysis.

### DNA damage measurements

nDNA and mtDNA damage were evaluated using a QPCR-based method as previously described [35] except that mtDNA damage was normalized to mtDNA copy number based on measurements obtained using the real-time method described above.

### mRNA levels

mRNA levels of five mitochondrial electron transport chain (ETC) complex subunits, including two mitochondria-encoded genes (*ctb-1* and *nd-5*) and three nucleus-encoded genes (*C34B2.8*, *D2030.4* and *K09A9.5*), were measured using real-time PCR. mRNA levels of the nuclear-encoded *polg-1* gene, which encodes the *C. elegans* mitochondrial DNA polymerase γ, were also measured. 250 ng of mRNA isolated from *C. elegans* as described above was converted to cDNA using the Qiagen Omniscript Reverse Transcription kit. Real time PCR was carried out with a 7300 Real Time PCR System as described above except that the extension temperatures were 62°C for *ctb-1* and *nd-5*, and 60°C for C34B2.8, D2030.4 and K09A9.5. The average mRNA fold change of each target gene was calculated by comparing the CT (cycle threshold) of the target gene to that of the housekeeping genes *cdc-42* and *pmp-3*[36]. Primers were based on the literature (*ctb-1*, *polg-1*, and *nd-5* from [28]) except that we used an annealing temperature of 62° rather than 60°C, were designed using Primer 3, or were recommended by Dr. Marni Falk (University of Pennsylvania: C34B2.8, D2030.4 and K09A9.5). The experiment was carried out twice for a total “n” of 3–5 except when the microarray samples were used in which case the “n” was 5–7 from 5–7 experiments. Unpublished primers were as follows: *cdc-42*, forward - 5’- GAG AAA AAT GGG TGC CTG AA-3’, reverse - 5’-CTC GAG CAT TCC TGG ATC AT-3’ (101 bp); pmp-3, forward - 5’- GTT CCC GTG TTC ATC ACT CAT-3’, reverse - 5’- ACA CCG TCG AGA AGC TGT AGA-3’ (115 bp); D2030.4, forward - 5’- GCG AGA TGA AGG CTA CTT GG-3’, reverse - 5’-GGT GCA TTT TGG GTT TGG-3’ (115 bp); K09A9.5, forward - 5’- AGT CAT CAT CAA GGC CAT CC-3’, reverse - 5’-TTG TTG GGA TGT CAA TAC CG-3’ (185 bp); C34B2.8, forward - 5’- CTT TTC CGA AGC TTG TCT GG-3’, reverse - 5’-CTT GGC CAA CAA TTT GAG C-3’ (197 bp). All samples were run in duplicate or triplicate and replicates were averaged prior to analysis.

### ATP assay

Steady-state ATP levels were determined by the luminescence level of the PE255 strain [20,37]. Luminescence was measured in a 96-well microplate reader (FLUOstar OPTIMA, BMG Labtech, Ortenberg, Germany) with approximately 300 nematodes per well (in 100 μl) in the visible spectral range between 300 and 600 nm (firefly luciferase typically emits at 550–570 nm). An automated dispenser delivered 50 μl of luminescence buffer to each well, consisting of citrate phosphate buffer pH 6.5, 0.1 mM D-luciferin, 1% DMSO and 0.05% triton-X (all final concentrations). Three separate experiments with 3–5 replicates total at each timepoint were conducted.

### Oxygen consumption measurement

Oxygen consumption over 2 minutes was measured in PE255 nematodes in an oxygen chamber (782 Oxygen Meter, Strathkelvin Instruments, North Lanarkshire, Scotland) as described [38]. *C. elegans* were washed with K medium and counted using a nematode sorter (COPAS, Union Biometrica, Holliston, MA). Three separate experiments with 2 replicate plates per timepoint per dose each were conducted (1–4 samples per plate were measured and averaged), resulting in a total n = 4–6. Each replicate contained 1000 nematodes for the 0, 3, and 24 h time points and 500 nematodes for the 48 h time point.

### Statistical analysis

All data except the microarray data were analyzed using Statview© for Windows (Version 5.0.1, SAS Institute Inc., Cary, NC). “Treatment,” “time,” and “experiment” were treated as independent variables in two- or three-way ANOVA analyses. When warranted based on initial ANOVA analyses, posthoc comparisons were carried out using Fisher’s Protected Least Significant Differences (FPLSD) test. Since oxygen consumption and ATP levels increased dramatically during larval development, and we wished to test for proportional differences based on treatment, we log-transformed those data prior to analysis. However, non-log-transformed data were plotted to avoid obscuring the large developmental changes that occurred. A p-value of less than 0.05 was considered statistically significant. Throughout the manuscript, error bars indicate the standard error of the mean.

## Results

### Transcriptomic response during and 3 h after UVC exposures

We examined the transcriptomic response to a UVC exposure protocol that results in high levels of mtDNA damage but allows for repair of the nDNA damage that is also induced [15]. Since EtBr, a specific inhibitor of mtDNA replication [39], exacerbates the response of *C. elegans* to such mtDNA damage [15], we also exposed half of the nematodes to EtBr. We considered the possibility that co-exposure to EtBr and UVC would lead to increased DNA damage compared to UVC alone due to photosensitization [40,41]; however, we did not detect any difference in DNA damage with EtBr co-exposure (Additional file 1: Figure S2). In a parallel experiment, we also measured mtDNA copy number throughout the exposure. The mtDNA copy number did not change in control nematodes, nor was there a marked change in mtDNA copy number due to UVC during the exposure (Additional file 1: Figure S2).

We sampled mRNA at 4 timepoints (3 h after the first UVC exposure or “-45 h”; 1 h prior to the second exposure or −25 h; 1 h prior to the final exposure, and 3 h after the final exposure and being placed on food: Figure 1). At each timepoint, nematodes were sampled that had been exposed to UVC, EtBr, both, or neither (controls). The “n” was 4–6 samples per timepoint per treatment, each generated from different experiments (separated in time).

Most differentially expressed transcripts (DETs) were associated with time. Three-factor ANOVA (time, UVC, EtBr with Benjamini-Hochberg correction) identified 10095 genes that showed differential expression over time, using a cut-off of p < 0.001 (which results in 10 differentially expressed genes expected by chance). Only 1447 DETs resulted from EtBr treatment, and 36 DETs resulted from UVC. At this cut-off only three genes demonstrated altered responses to EtBr based on time (i.e., a time x EtBr interaction), six genes demonstrated a time-dependent response to UVC, and no genes were identified for which the response to UVC was dependent on the presence of EtBr or EtBr and time. PCA results also indicated a very strong effect of time. PCA and detailed ANOVA results for the global dataset are presented in Additional file 1: Figure S3. Since we were most interested in the combined effect of prolonged inhibition of mtDNA replication and mtDNA damage, and since the presence of food is likely to alter mitochondrial function and response to stressors, we also carried out ANOVA on the 3 h timepoint samples only. However, we found no significant (p < 0.05) UVC x EtBr interactions for any genes at 3 h.

**Figure 2:**
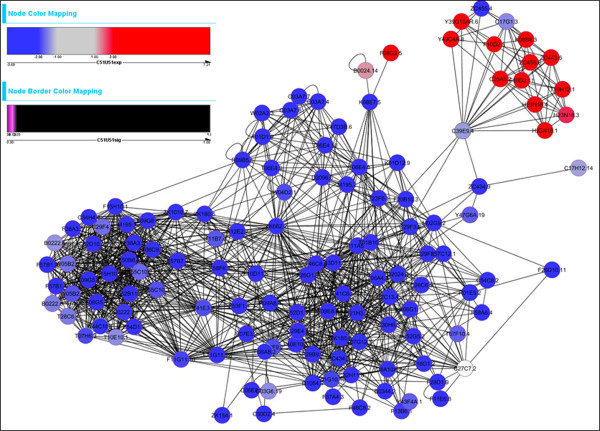
**At 3 h, the combination UVC + EtBr treatment resulted in dramatic changes in expression of networked genes.** The blue genes are downregulated after the combination treatment and comprise genes belonging to body morphogenesis and other developmental processes. Most red genes (upper right cluster) are part of lipid and carbohydrate metabolic processes.

At 3 h, all three treatments resulted in many DETs associated with many developmental processes. Shown in Figure 2 is one of the most altered gene networks (“neighborhoods”) identified by jActiveModules at 3 h when comparing the combination treatment to control nematodes; BiNGO-based GO enrichment analysis identified developmental processes as a top altered process. Similar GO results were obtained using GeneSpring to characterize DETs detected by ANOVA. Furthermore, the combination treatment led to greater alterations in expression of developmental genes compared to either treatment alone (e.g., Additional file 1: Figure S4). This is consistent with our previous observation of developmental delay after both treatments, which was strongest in the combination [15].

**Figure 3:**
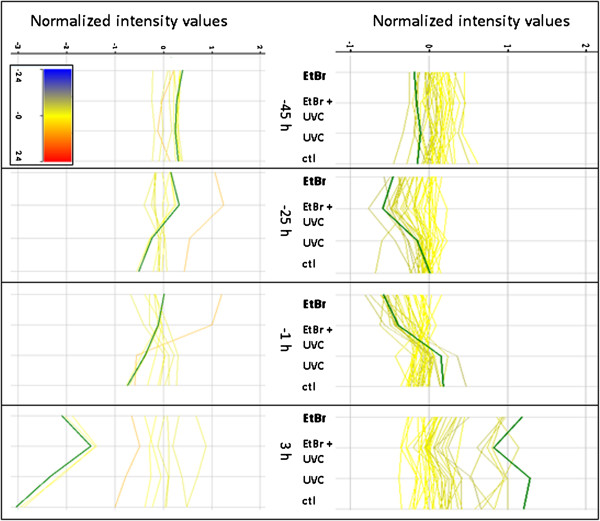
**EtBr and UVC exposures resulted in decreased expression of many oxidative phosphorylation genes (top panel; *sucg-1 *highlighted in green), but increased expression of some glyoxylate genes (bottom panel; *icl-1 *highlighted in green).** Red-blue scale coloration is based on comparison to mRNA levels in control samples at −45 h. Normalized intensity values are on a binary log scale (i.e. “1” indicates a 2-fold change, “2” a 4-fold change, etc.). n = 4–6.

We hypothesized that persistent mtDNA damage would lead to reduced (via damage-mediated inhibition of the mitochondrial RNA polymerase) or upregulated (compensatory) expression of genes coding for mitochondrial proteins, both from the mitochondrial and nuclear genomes. Using published lists of mitochondrial genes [42], we found that at timepoints later than 3 h, some transcripts for oxidative phosphorylation decreased in the presence of EtBr, and this effect was often exacerbated by UVC (Figure 3). Furthermore, several genes in the glyoxylate pathway were induced by EtBr and EtBr + UVC at several timepoints; many of these same transcripts were also decreased in abundance across treatments at 3 h (after food addition) (Figure 3). We observed no statistically significant differences in mitochondrial-encoded transcripts (not shown), but since these are poorly represented on the platform that we used, we also carried out additional real time PCR-based analyses (see below).

Based on the role of mitochondrial fusion, fission, and autophagy in responding to UVC-induced mtDNA damage [15], we examined expression specifically of genes in these pathways. We observed a small increase in expression of some autophagy genes at −25 and −1 h, along with an inhibition of the decrease observed in many autophagy genes at 3 h, after food was available (Additional file 1: Figure S5). There was either no change or a small increase in fusion and fission genes after EtBr and EtBr +UVC, only at 3 h (Additional file 1: Figure S5).

While of less relevance for this manuscript, the transcriptomic responses to EtBr and UVC alone are interesting in their own right. The DETs (defined liberally as p < 0.05 and fold-change >1.2, based on Rosetta Resolver values) for each pairwise treatment comparison at each timepoint are provided in Additional file 3. The short-term (−45 h) response was stronger in terms of number of regulated genes for UVC than EtBr, but the reverse was true thereafter. This suggests that UVC led to a more robust signaling response, but EtBr altered more biological processes over time or that the response was slower due to the kinetics of uptake of EtBr. The most altered gene ontologies observed for UVC exposure were stress response and aging. EtBr treatment alone altered expression of many development-related genes (as described earlier) but also led to a dramatic induction of many xenobiotic metabolism genes, in particular cytochrome P450s (some of which were induced 50 to 100-fold: Additional file 1: Figure S6), but also including p-glycoprotein and glutathione S-transferase genes (Additional file 3). Some xenobiotic metabolism genes, however, were down-regulated by EtBr (e.g., *cyp-35A3*, *ugt-37*, and *gst-25*; Additional file 3). Finally, of all DNA repair genes that we identified previously [34], only one was strongly upregulated by any treatment at any time: *pme-4* (Additional file 1: Figure S7).

We next carried out additional experiments to test whether mitochondrial function was in fact altered, as suggested by the transcriptomic data, and whether the mild alterations in levels of mtDNA- and nuclear genome-encoded genes would persist to later timepoints.

### mtDNA damage was persistent to the L4 stage (48 h timepoint)

**Figure 4:**
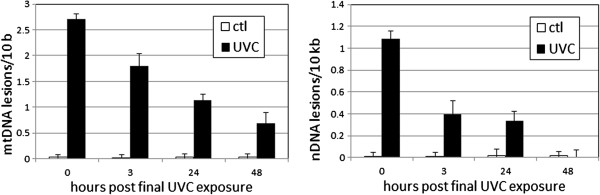
**Exposure to UVC resulted in persistent mtDNA damage in larval *C. elegans.*** Main effects of treatment, time, genome, and all interactions were significant (p ≤ 0.0002). At 0, 3, and 24 h after the last exposure, DNA damage was statistically significant in both genomes. At 48 h, DNA damage was detected in mtDNA (p = 0.0009) but not nDNA (p = 0.82). Note different y-axis scales. n = 4–11 in two experiments.

Immediately (0 h) after the third UVC exposure, 2.7 ± 0.1 mitochondrial and 1.1 ± 0.1 nuclear DNA lesions per 10 kilobases were detected in larval *C. elegans* (Figure 4). nDNA damage dropped to 0.4 ± 0.1 at 3 h post-exposure, and to 0.0 ± 0.1 in 48 h. The mtDNA lesions, in contrast, persisted at 1.8 ± 0.2 and 0.7 ± 0.2 at 3 and 48 h, respectively. These results suggest a dilution of the UVC-induced DNA damage due to replication of the nuclear and mitochondrial genomes throughout *C. elegans* development, since the kinetics of photodimer removal in nuclear and mitochondrial DNA in *C. elegans* would not explain this rate of reduction [15,19,34].

### Measurement of mtDNA and nDNA copy number throughout development

We used RT-PCR to establish a baseline of mtDNA and nDNA copy number in N2 nematodes (Table 1). As previously observed [9,28], mtDNA copy number increased significantly late in development. In contrast to those previous reports, we saw only a slight increase in mtDNA copy number in early larval stages that did not keep pace with nDNA replication, resulting in a >10-fold decrease in mtDNA:nDNA ratio from freshly laid eggs to hatched L1s. This ratio then roughly doubled by adulthood. These studies were performed on eggs laid on plates during 1 h, so that “0 h” refers to eggs frozen within 1 h of being laid. Since our subsequent experiments were carried out on nematodes that were raised from eggs that were age-synchronized via bleach-sodium hydroxide isolation of eggs and liquid hatch, we also compared mtDNA copy number after one or two days without food in M9 medium (comparable to the conditions used for the serial UVC exposure). The egg prep resulted in a decrease of ~50% in mtDNA copy number in the growth-arrested L1s, but copy number returned to normal by the L3 stage (data not shown; also compare Table 1 with Additional file 1: Figure S2).

**Table 1:** **Nuclear and mitochondrial DNA copy number in wildtype (N2)*C. elegans*raised ay 20 C, starting from eggs laid on k agar plates, ± standard error of the mean**

**Developmental stage**	**nDNA copy number**	**mtDNA copy number (x10**^**4**^**)**	**mtDNA:nDNA ratio**
Egg, 0 h (n=6)	92±36	4.12±1.30	948±498
Hatch, 13 h (n=15)	890±23	6.26±0.16	71±3
Mid-L1, 21 h (n=7)	1155±57	9.96±0.58	87±4
Mid-L2, 33.5 h (n=7)	1492±119	14.00±1.45	94±7
L2/L3, 38 h (n=8)	2000±140	20.17±1.77	101±4
Early L3, 41 h (n=8)	2856±235	29.23±2.72	103±5
Late L3, 44 h (n=8)	3242±245	38.63±3.18	119±6
L3/L4, 47 h (n=7)	3967±273	48.39±3.55	123±6
Early L4, 50.7 h (n=8)	4805±205	56.12±4.02	116±5
Late L4, 54.3 (n=12)	5552±325	73.99±7.43	131±7
L4/young adult, 58 h (n=10)	6496±228	102.77±4.81	159±8
Early young adult, 66 h (n=12)	7430±550	126.49±12.18	174±13
Late young adult, 66 h (n=11)	7737±310	123.57±12.15	157±12
Adult, 70 h (n=12)	9456±315	140.72±6.10	152±10

“0 h” refers to eggs laid within a 1 h period and immediately collected frozen. “n” indicates the number of individual eggs or nematodes lysed and separately analyzed.

### UVC exposure affected mtDNA copy number but not nDNA copy number

**Figure 5:**
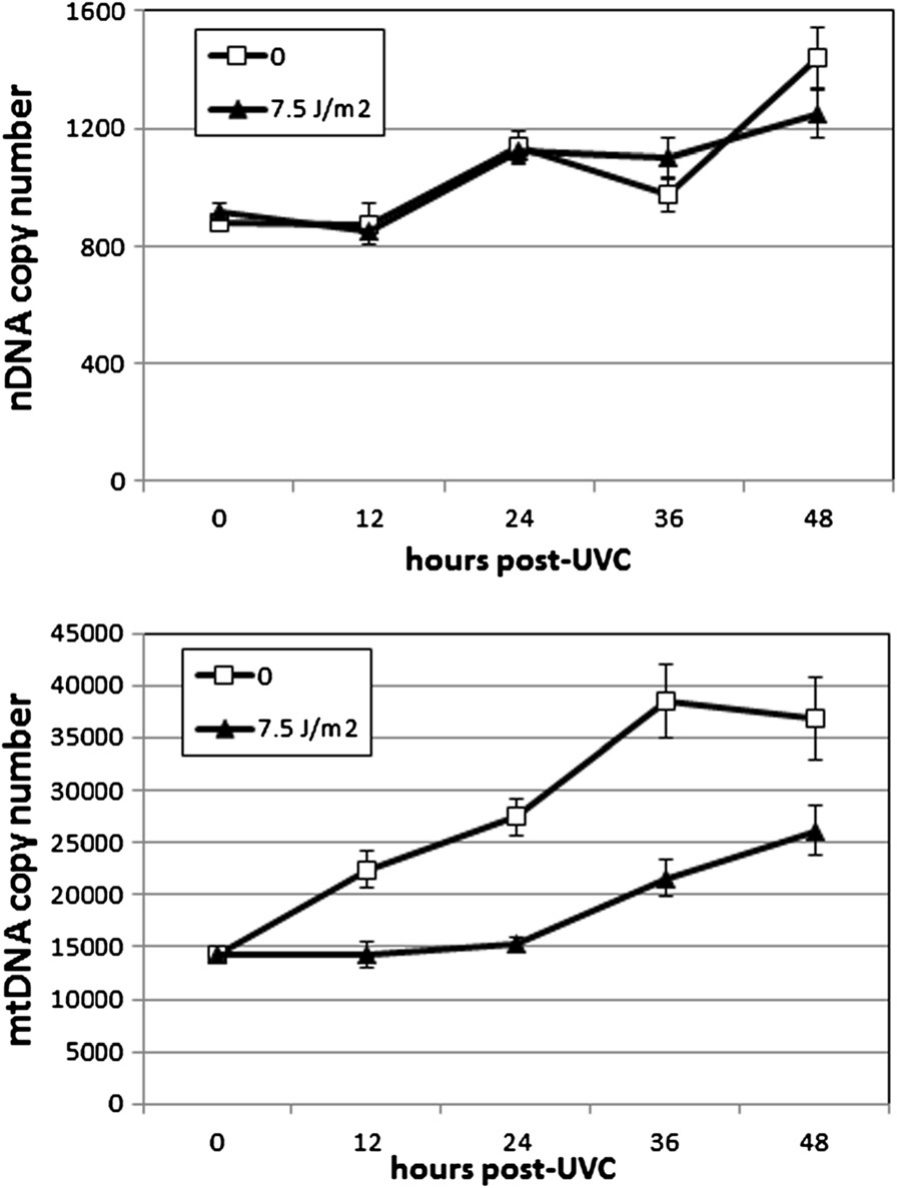
**Exposure to UVC did not cause a detectable change in nDNA copy number, but decreased mtDNA copy number at later time points.** Effect on nDNA: p = 0.82 for main effect of UVC, p = 0.37 for interaction of UVC with time, p < 0.0001 main effect of time. Effect on mtDNA: p < 0.0001 for main effects of UVC and time, p = 0.15 for interaction. n = 8–20 (3 or 4 separate experiments).

Because somatic cell division and development are invariant in *C. elegans*[31], nDNA copy number serves as a proxy for developmental stage. Despite the development-related transcriptomic responses we observed and the mild developmental delay that we previously documented at this dose of serial UVC [15], we did not detect a reduction in nDNA copy number during this experiment (p = 0.82 for main effect of treatment, p < 0.0001 for main effect of time; p = 0.37 for interaction; Figure 5). Nonetheless, preliminary experiments suggested that adult egg reproduction (i.e., at later timepoints) was affected by the UVC treatment.

In contrast, and as hypothesized based on the *in vitro* ability of UVC-induced photodimers to inhibit DNA polymerase γ [16], mtDNA copy number was decreased at all later timepoints (p < 0.0001 for main effects of time and treatment; p = 0.048 for interaction; timepoints past 0 h p < 0.05 UVC vs control by FPLSD; Figure 5).

These experiments were carried out in the PE255 strain, in order to permit comparison to the ATP data derived from that strain (see below). However, we observed similar results (no effect on nDNA copy number, decreased mtDNA copy number) in preliminary experiments with N2 and *glp-1* strains as well (data not shown).

### UVC exposure altered expression of mtDNA- and nDNA-encoded mRNAs

We also hypothesized that UVC-induced mtDNA damage would inhibit the mitochondrial RNA polymerase [17], resulting in a decrease in mtRNAs. Because our microarray platform has few probes for mtDNA-encoded genes, we examined those samples using RT-PCR analysis. At 3 h, but not earlier, mRNA levels for the mtDNA-encoded genes *ctb-1* and *nd-5* were decreased ~50% after exposure to UVC or UV + EtBr, without a change in mRNA levels for 4 nDNA-encoded mRNAs (*C34B2.8*, *D2030.4* and *K09A9.5*, coding for ETC (Complex I) components, and *polg-1*, coding for the mitochondrial DNA polymerase γ) (Additional file 1: Figure S8). However, formal statistical comparisons of each gene at each timepoint could not be carried out due to the lack of a significant time x treatment x gene interaction.

**Figure 6:**
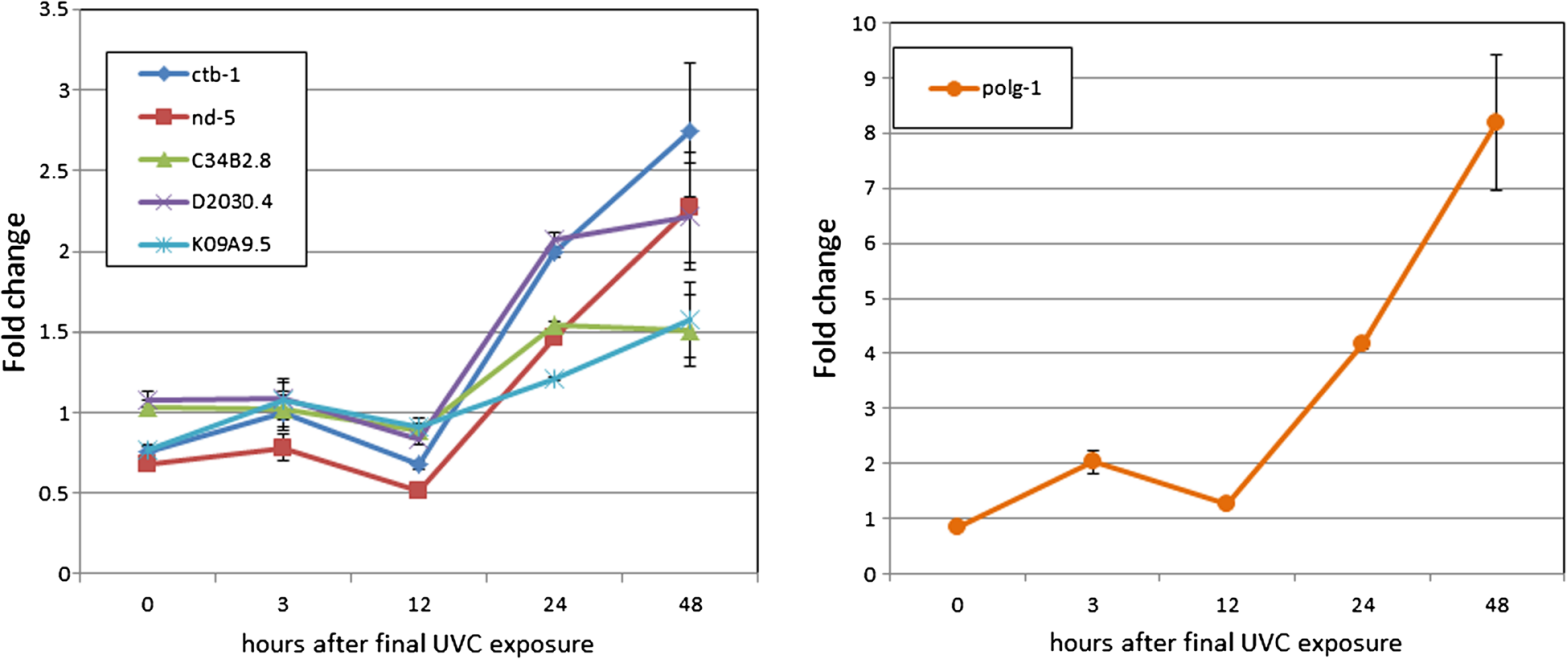
**Exposure to UVC reduced *ctb-1 *and *nd-5 *expression at early timepoints, but resulted in higher levels of all mRNAs at later times (wild-type N2 strain).***ctb-1* and *nd-5* are mtDNA-encoded, and C34B2.8, D2030.4, K09A9.5, *polg-1* are nDNA-encoded, mitochondrial proteins. *polg-1* is graphed separately to avoid obscuring the smaller fold-changes observed in the other genes; note different y-axes. p < 0.0001 for all main and interactive effects, 3 factor ANOVA. Fold change is relative to the mRNA of the same gene at the same time without UVC exposure. n = 3–6.

Next, to determine whether this change was persistent through development, we repeated the experiments and measured mRNA levels for the same genes at 0, 3, 12, 24, and 48 h post-exposure. We observed lower amounts of the mtDNA-encoded mRNAs at 0–12 h (Figure 6). However, we also detected a significant increase in mRNA levels for all of the examined transcripts at 24 and 48 h, including an 8-fold induction of *polg-1* at 48 h post-exposure (Figure 6). Finally, we replicated this experiment in the PE255 strain, with somewhat different results. In the PE255 nematodes, the mtDNA and most nDNA-encoded ETC component mRNAs were generally lower at nearly all timepoints after UVC; only *polg-1* was induced, but to a lesser extent than in the N2 nematodes (Additional file 1: Figure S9).

### UVC exposure resulted in a delayed decrease in steady-state ATP level

**Figure 7:**
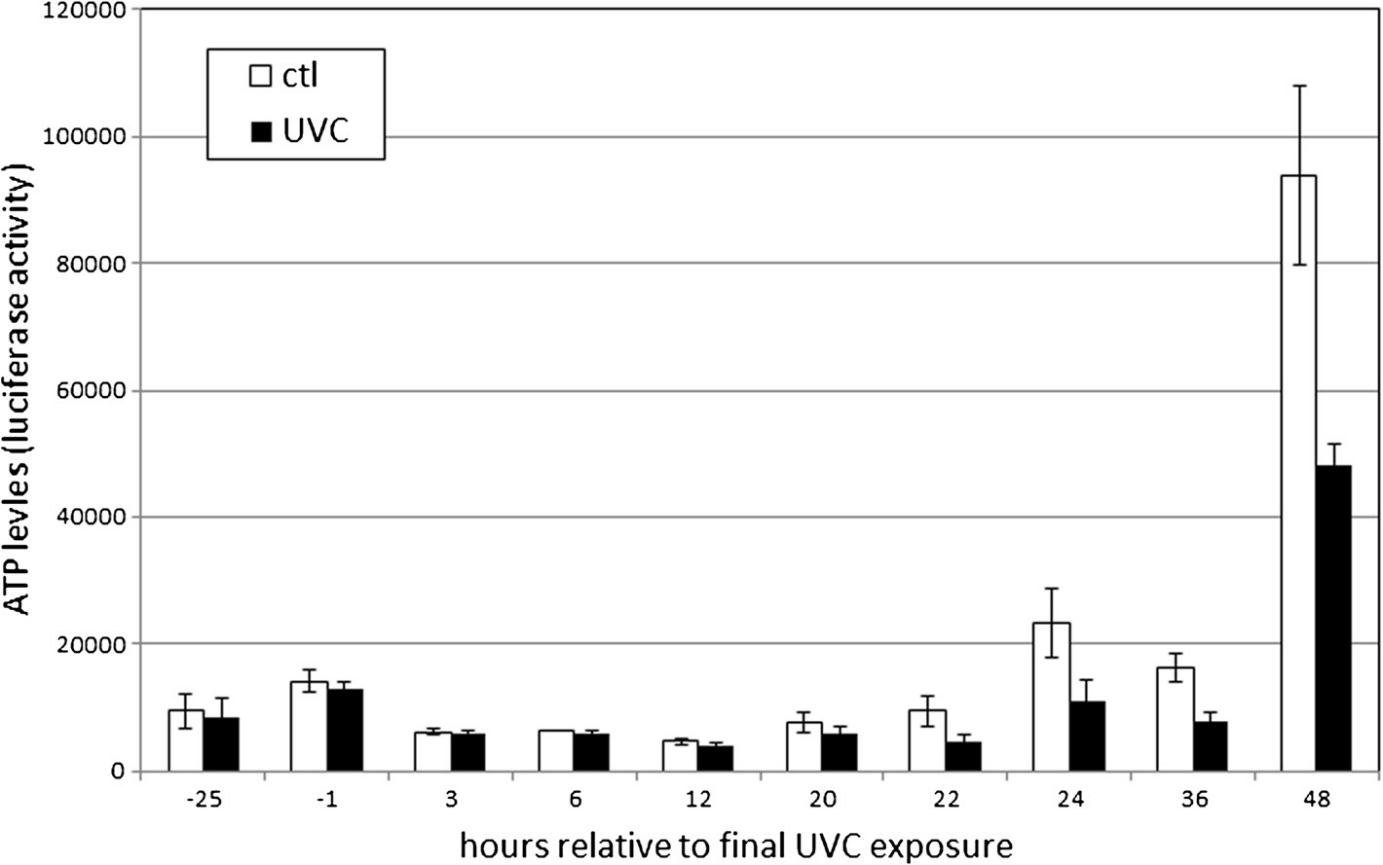
**Exposure to UVC reduced ATP levels at later larval stages in developing *C. elegans*.** The main effects of UVC and time were significant (p = 0.003 and p < 0.0001 respectively), but their interaction was not, precluding comparisons at specific timepoints. n = 5–7 separate experiments; 5 separate measurements per experiment were pooled for each “n”.

To test if altered mtDNA and mtRNA levels were associated with altered mitochondrial energy production, we measured ATP levels *in vivo* using a well-validated [20,43] transgenic strain expressing a luciferase gene. ATP levels were similar in control and UVC-exposed nematodes until ~24 h after the UVC exposure, despite a ~50% drop in the 3 h after the nematodes were plated on food plates (Figure 7). As observed previously using traditional methods for ATP analysis [44], ATP levels increased significantly during larval development. The UVC treatment resulted in a ~50% decrease in ATP levels at 24, 36, and 48 h post-exposure, when ATP levels were rising significantly (Figure 7) (p = 0.003 and p < 0.0001 for main effects of UVC and time).

### UVC exposure resulted in decreased oxygen consumption

**Figure 8:**
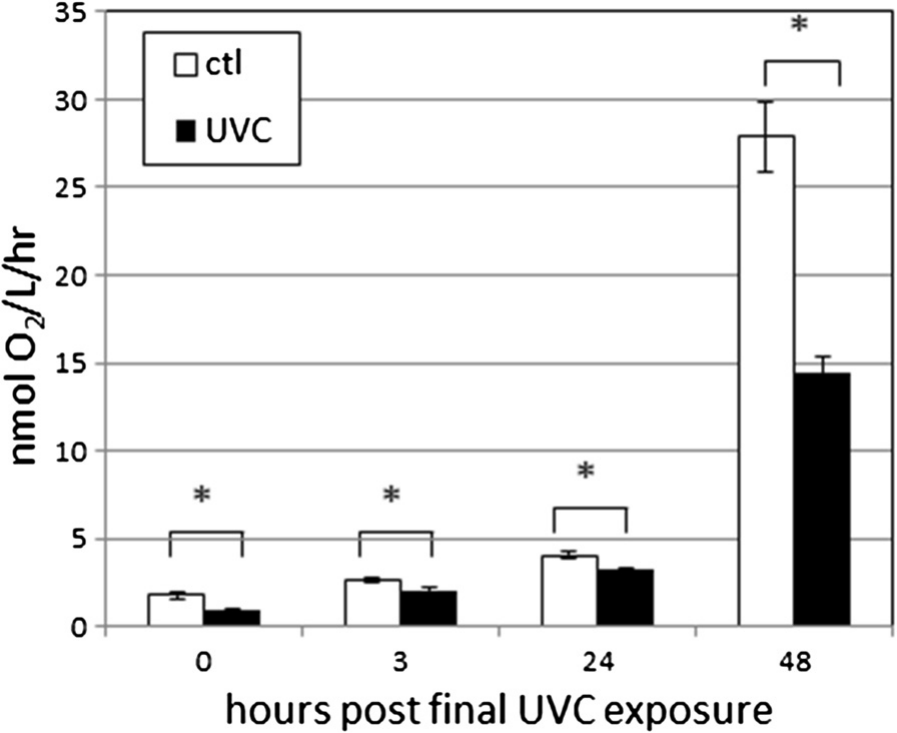
**Exposure to UVC reduced oxygen consumption in larval *C. elegans.*** n = 4–6 in 3 experiments. The effects of time and UVC and their interactions were all significant at p < 0.0001 (ANOVA); all FPLSD comparisons for the effect of UVC at individual timepoints were significant at p ≤ 0.001 (indicated by asterisks).

Since ATP can also be produced anaerobically and is a function of both production and use, steady-state ATP levels are an indirect readout of oxidative phosphorylation function. Therefore, to complement the ATP data, we also measured oxygen consumption in PE255 nematodes. Oxygen consumption, unlike ATP levels, did not drop between 0 and 3 h post-exposure (Figure 8), suggesting that the drop in ATP after addition of food resulted from a higher flux of ATP rather than an overall decrease in energy production. Consistent with previous research [44], we observed a strong increase in oxygen consumption during larval development. The UVC exposure resulted in a statistically significant (p ≤ 0.001, FPLSD) decrease in oxygen consumption at all timepoints. However, the degree of decrease varied (p < 0.0001 for time x treatment interaction, ANOVA): ~50% decrease at 0 and 48 h post-exposure, but ~20% at 3 and 24 h (Figure 8).

## Discussion

Previous work has demonstrated that mtDNA damage generated by exposure to alkylating and oxidative agents can cause mitochondrial dysfunction in a cell culture system [14]. However, such damage is generally repairable in mtDNA [3,45]. In contrast, UVC-induced DNA damage is not repaired in mtDNA in *C. elegans*[19], although it can be slowly removed [15]. Here, we report on the effect of a serial UVC exposure, which results in highly persistent mtDNA damage, on genome-level transcription and mitochondrial function.

The strong overall effect of time on our transcriptomic profiles is not surprising, since the transcriptomic response to starvation in L1s is established within 6 h, and the response to food addition is established even more quickly (by 3 h) due to RNA polymerase II accumulation in the promoters of growth-related genes [46]. However, we were surprised not to observe a strong mitochondria-related transcriptomic response to serial UVC alone or in combination with the mtDNA intercalator EtBr. At the timepoints examined, the transcriptomic response to persistent mtDNA damage (from the UVC) and inhibition of mtDNA replication (from the EtBr) was mild and not very different than EtBr alone. Taken together, these results suggest that *C. elegans* lacks a way to specifically identify and respond transcriptionally to mtDNA damage, that this response is inactive at that time in development, or that any such response is very limited.

Although the transcriptomic response to EtBr by itself was quite robust, it did not include genes that are obviously related to mtDNA maintenance. Rather, the response was dominated by induction of “xenobiotic metabolism” (including cellular efflux pumps) as well as nuclear hormone receptor genes. The category of “xenobiotic metabolism,” however, should be treated with caution since the actual substrate specificity of the corresponding enzymes has generally not been tested. Nonetheless, the degree of induction is remarkable, and adds to the growing literature on the ability of this nematode to transcriptionally modulate the metabolic response to xenobiotics [47-49].

UVC, EtBr, and the combination acted to inhibit development-related transcriptomic changes associated with food addition, and also altered the mRNA levels for genes involved in energy metabolism both during starvation and after food addition. This is consistent with the induction of glycolysis genes in human cells depleted of mtDNA [50]. It is also consistent with our other results that showed decreased ATP levels, oxygen consumption, and mtDNA copy number, since all of these typically increase during development. We note that we measured ATP levels and oxygen consumption only in PE255 nematodes, and the difference in UVC-induced mRNA levels indicates a need for caution in extrapolating results between PE255 and N2 nematodes. However, the developmental patterns that we observed were similar to those previously published for N2 nematodes, suggesting that overall mitochondrial function is likely similar in N2 and PE255 nematodes.

It is interesting that the observed decreases in mitochondrial function were not associated with a decrease in nDNA copy number. This suggests that development itself, as measured by cell division (which occurs according to an invariant pattern in *C. elegans*), was not significantly hindered. This conclusion is also supported by our previous observation of only a very slight delay in development at this dose of serial UVC [15]. This is surprising given the documented ability of mitochondrial dysfunction to inhibit larval development in *C. elegans*[9,51,52]. We propose two possibilities to explain this: first, as suggested by previous work, that the developmental delay results not so much from mitochondrial dysfunction *per se*, but rather from a signaling event that presumably was not activated at this level of damage [52,53]; second, that the threshold of mitochondrial dysfunction required to hinder development was not reached. These hypotheses, as well as an exploration of effects in adults, are important areas of future research.

We hypothesized that since the DNA damage was repaired in nDNA but not mtDNA, transcription of ETC components would be imbalanced, resulting in mitochondrial dysfunction. While the decreases in mRNA levels that we observed at early times were not enormous (maximally 50%), we note that in human cells, mtRNA represents between 5% and 30% of total cellular RNA [54], suggesting that production of high levels is important. However, there was no induction of *hsp-6* and *hsp-60*, which respond to imbalance of ETC proteins [55]. This suggests the possibility of retrograde signaling that permits the organism to maintain an appropriate balance of ETC proteins. Similarly, there was not a strong transcriptomic signature indicative of oxidative stress (e.g., induction of *gst-4* or *gcs-1*: [56,57]), as might be expected if there were significant ETC dysfunction [58]. The lack of induction of these and other common general stress-response pathways also suggests that, although UVC is not entirely specific for nucleic acids, damage to other cellular macromolecules was not widespread. Overall, the relatively mild response to persistent mtDNA damage suggests that *C. elegans* has a significant ability to maintain mitochondrial function despite such damage, as we recently observed is true for primary human fibroblasts [59].

The significant induction in *polg-1* and ETC mRNAs at 24 and 48 h post-exposure may also explain in part the nematodes’ ability to recover from damage, and to begin replenishing the mtDNA population by 48 h. It may be that *polg-1* was not induced at early timepoints because of the relatively low dependence on mitochondrial function exhibited by *C. elegans* at those developmental stages. That insensitivity has been previously demonstrated by studies showing that mitochondrial dysfunction results in developmental arrest at the L3 or L4 stage, not earlier [9,51,52], and that *C. elegans* lacking both copies of *polg-1* are able to survive to late larval stages and even in some cases early adulthood [28]. Similarly, the induction of ETC genes supports an adaptive response, and is consistent with the ability to tolerate and transcriptionally compensate for mtDNA depletion previously observed in HeLa cells [60].

A fuller understanding of the basic biology of mtDNA maintenance in *C. elegans* will help elucidate a more complete understanding of the replicative and transcriptional response to such damage in this organism. Mitochondrial biogenesis is an important response to mitochondrial stress or dysfunction in mammalian cells [61], and our mRNA induction data supports such a response in *C. elegans*. However, mitochondrial biogenesis *per se* has not been described in *C. elegans*. In addition, although there are many similarities with mammalian mtDNA, there are also differences. For example, TFAM has not been found in *C. elegans*—perhaps because if present, it may not have a transcriptional role, as appears to be the case in yeast [62]. It is also interesting that in *C. elegans* there are apparently no genes on the light strand, raising questions about how transcription and replication are coupled in this species, since light strand transcription is the mechanism for priming mtDNA replication in humans [63,64].

## Conclusions

In summary, our results support the hypothesis that early life exposure to persistent mtDNA damage can lead to later life mitochondrial dysfunction. However, they also highlight the ability to compensate for or respond to such damage *in vivo*. Some of this capacity is likely the result of the ability to clear such damage via mitochondrial dynamics and autophagy [15]. An important direction of future research will be to investigate how deficiencies in those processes—which are observed in the human population—will affect the response to such damage.

## Competing interests

The authors declare that they have no competing interests.

## Authors’ contributions

MCKL, JPR, ITR, ASB, and AQJ carried out measurements of mtDNA damage, mtDNA and nDNA copy number, mRNA levels by RT-PCR, oxygen consumption, and ATP levels. AJB and TLC carried out microarray experiments. MCKL, JPR and JNM participated in study design and coordination; MCKL and JNM drafted the manuscript. All authors read and approved the final manuscript.

## Pre-publication history

The pre-publication history for this paper can be accessed here:

http://www.biomedcentral.com/2050-6511/14/9/prepub

## Supplementary Material

Additional file 1: Figure 1 The *C. elegans* mitochondrial genome, produced with Organellar GenomeDraw (Lohse et al., 2007). The blue arrow indicates the direction of transcription (Okimoto et al., 1992). mRNA levels of the two genes highlighted in purple were measured using RT-PCR. **Figure 2.** Exposure to EtBr (5 μg/mL) did not result in measureable DNA damage (measured 1 h after the third UVC exposure) in either genome, nor did it exacerbate UVC-induced DNA damage in either genome (p > 0.05 for main effect of EtBr and interaction term, 2 factor ANOVA). The exposures did not result in a marked change in mtDNA:nDNA ratio (bottom panel); time (p = 0.50), treatment (p = 0.91) and the interaction term (p = 0.89) were all statistically insignificant. Therefore, we could not make direct within-time comparisons by FPLSD. n = 4-8 per bar. **Figure 3.** Time and EtBr were the major drivers of differential gene expression by ANOVA and PCA. Even at the least stringent p-value, where 748 differentially expressed genes are expected to be identified by chance, no genes were differentially regulated in response to UVC in a way that was modulated by EtBr or EtBr and time. In the PCA (three views of same plot), the x-axis (component 1) explains 52% of variability, the y-axis 21%, and the z-axis 14%. Blue indicates -45 h, red -25 h, maroon -1 h, and grey 3 h. Diamonds indicate control samples, circles UVC, squares EtBr, and triangles UVC + EtBr. Analyses performed with GeneSpring. **Figure 4.** At 3 h, the combination treatment led to additional effects compared to EtBr alone. Development is the most-altered gene ontology; differences were also observed in transcription, protein catabolism, and organellar organization. Blue indicates higher expression in the combination than in EtBr alone. **Figure 5.** Some autophagy genes were induced by EtBr and UVC at later timepoints (top panel; *lgg-2* highlighted). No changes were observed in fusion or fission genes (bottom panel; *eat-3* highlighted). Red-blue scale coloration is based on comparison to mRNA levels in control samples at -45 h. Normalized intensity values are on a binary log scale (i.e. “1” indicates a 2-fold change, “2” a 4- fold change, ETC). n = 4-6. **Figure 6.** Many cytochrome P450 genes were upregulated by exposure to ethidium bromide; shown are genes upregulated by EtBr and fitting the GO term “monoxygenase activity.” The genes shown are *cyp-35B3* (highlighted in green), *cyp-13A7*, *cyp-35A5*, *cyp-35B1*, *cyp- 33C3*, *cyp-33C6*, *cyp-33C7*, *cyp-33D3*, *cyp-35B2*, *cyp-35A1*, *cyp-33C5*, and *cyp-33C4*. Red-blue scale coloration is based on comparison to mRNA levels in control samples at -45 h. Normalized intensity values are on a binary log scale (i.e. “1” indicates a 2-fold change, “2” a 4-fold change, ETC). n = 4-6. **Figure 7.** There is little change in expression of known DNA repair genes (from Boyd et al., 2010) either with time or treatment, with the exception of *pme-4* (highlighted). Red-blue scale coloration is based on comparison to mRNA levels in control samples at -45 h. Normalized intensity values are on a binary log scale (i.e. “1” indicates a 2-fold change, “2” a 4-fold change, ETC). n = 4-6. **Figure 8.** Effect of exposure to UVC, EtBr or both on mRNA levels for mtDNA-encoded (*ctb-1*, *nd-5*) and nDNA-encoded (C34B2.8, D2030.4, K09A9.5, *polg-1*) mitochondrial proteins. The legend is the same for all graphs. p < 0.05 for main effects of time, treatment, and genome, and genome x time interactions. Fold change is relative to the mRNA of the same gene at the same time without UVC exposure. n = 5-7 (samples derived from microarray experiment exposures, including additional samples not used for microarray). **Figure 9.** Effects of exposure to UVC in the PE255 strain. *polg-1* is graphed separately for consistency with Figure 6. *ctb-1* and *nd-5* are mtDNA-encoded, and C34B2.8, D2030.4, K09A9.5, *polg-1* are nDNA-encoded, mitochondrial proteins. The effect of UVC and gene (p = 0.016 and p < 0.0001 respectively) were significant, as was the interaction of UVC with gene (p = 0.003). No other main or interactive effects were significant (p > 0.05, 3 factor ANOVA). Comparisons at specific times could not be made due to the lack of significant interactions involving time. Gene-by-gene comparisons (across time) by FPLSD indicated that *polg-1* behaved differently than all other genes (p ≤ 0.003), and *nd-5* was distinct from K09A9.5 (p = 0.02). Fold change is relative to the mRNA of the same gene at the same time without UVC exposure n = 3-6. Click here for file

Additional file 2 **This file contains the “Union4” interactome described in the text (Methods section), containing 14334 nodes and 346484 edges.** It is in the Cytoscape-compatible .sif file format. Click here for file

Additional file 3 This file contains differentially expressed transcripts (defined as fold-change >1.2, p < 0.05 based on Rosetta Resolver analysis) for all pairwise treatment comparisons at the 3 h timepoint. Click here for file
