# Supplementary material for: Effects of early life exposure to ultraviolet C radiation on mitochondrial DNA content, transcription, ATP production, and oxygen consumption in developing Caenorhabditis elegans
Source: BMC Pharmacol Toxicol. 2013 Feb 4;14:9. doi: 10.1186/2050-6511-14-9 (PMC3621653; doi:10.1186/2050-6511-14-9)
Supplement: Additional file 1: Figure 1 — The C. elegans mitochondrial genome, produced with Organellar GenomeDraw (Lohse et al., 2007). The blue arrow indicates the direction of transcription (Okimoto et al., 1992). mRNA levels of the two genes highlighted in purple were measured using RT-PCR. Figure 2. Exposure to EtBr (5 μg/mL) did not result in measureable DNA damage (measured 1 h after the third UVC exposure) in either genome, nor did it exacerbate UVC-induced DNA damage in either genome (p > 0.05 for main effect of EtBr and interaction term, 2 factor ANOVA). The exposures did not result in a marked change in mtDNA:nDNA ratio (bottom panel); time (p = 0.50), treatment (p = 0.91) and the interaction term (p = 0.89) were all statistically insignificant. Therefore, we could not make direct within-time comparisons by FPLSD. n = 4-8 per bar. Figure 3. Time and EtBr were the major drivers of differential gene expression by ANOVA and PCA. Even at the least stringent p-value, where 748 differentially expressed genes are expected to be identified by chance, no genes were differentially regulated in response to UVC in a way that was modulated by EtBr or EtBr and time. In the PCA (three views of same plot), the x-axis (component 1) explains 52% of variability, the y-axis 21%, and the z-axis 14%. Blue indicates -45 h, red -25 h, maroon -1 h, and grey 3 h. Diamonds indicate control samples, circles UVC, squares EtBr, and triangles UVC + EtBr. Analyses performed with GeneSpring. Figure 4. At 3 h, the combination treatment led to additional effects compared to EtBr alone. Development is the most-altered gene ontology; differences were also observed in transcription, protein catabolism, and organellar organization. Blue indicates higher expression in the combination than in EtBr alone. Figure 5. Some autophagy genes were induced by EtBr and UVC at later timepoints (top panel; lgg-2 highlighted). No changes were observed in fusion or fission genes (bottom panel; eat-3 highlighted). Red-blue scale coloration is based on [file 2050-6511-14-9-S1.pdf]

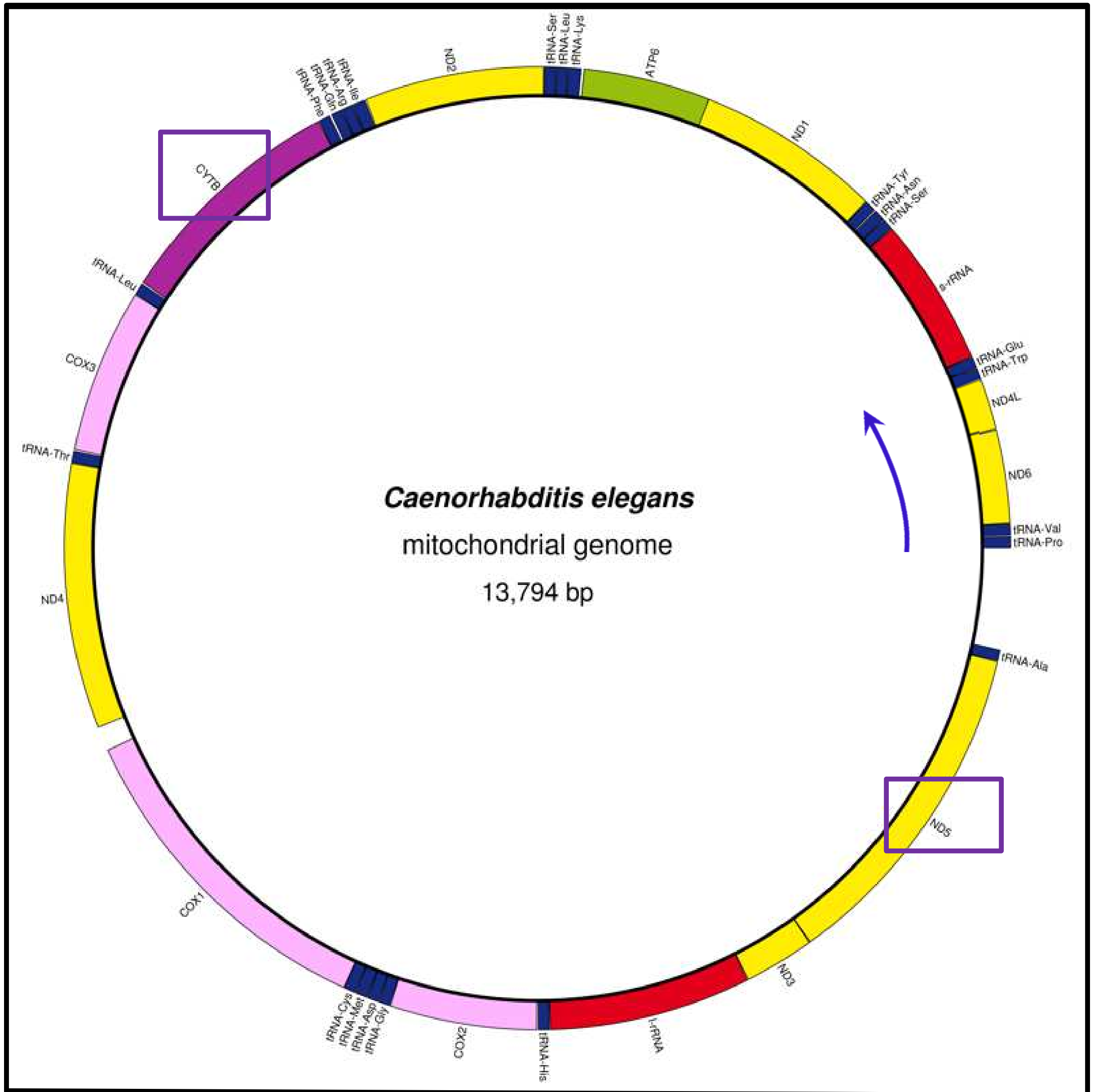

Additional Figure 1. The *C. elegans* mitochondrial genome, produced with Organellar GenomeDraw (Lohse et al., 2007). The blue arrow indicates the direction of transcription (Okimoto et al., 1992). mRNA levels of the two genes highlighted in purple were measured using RT-PCR.

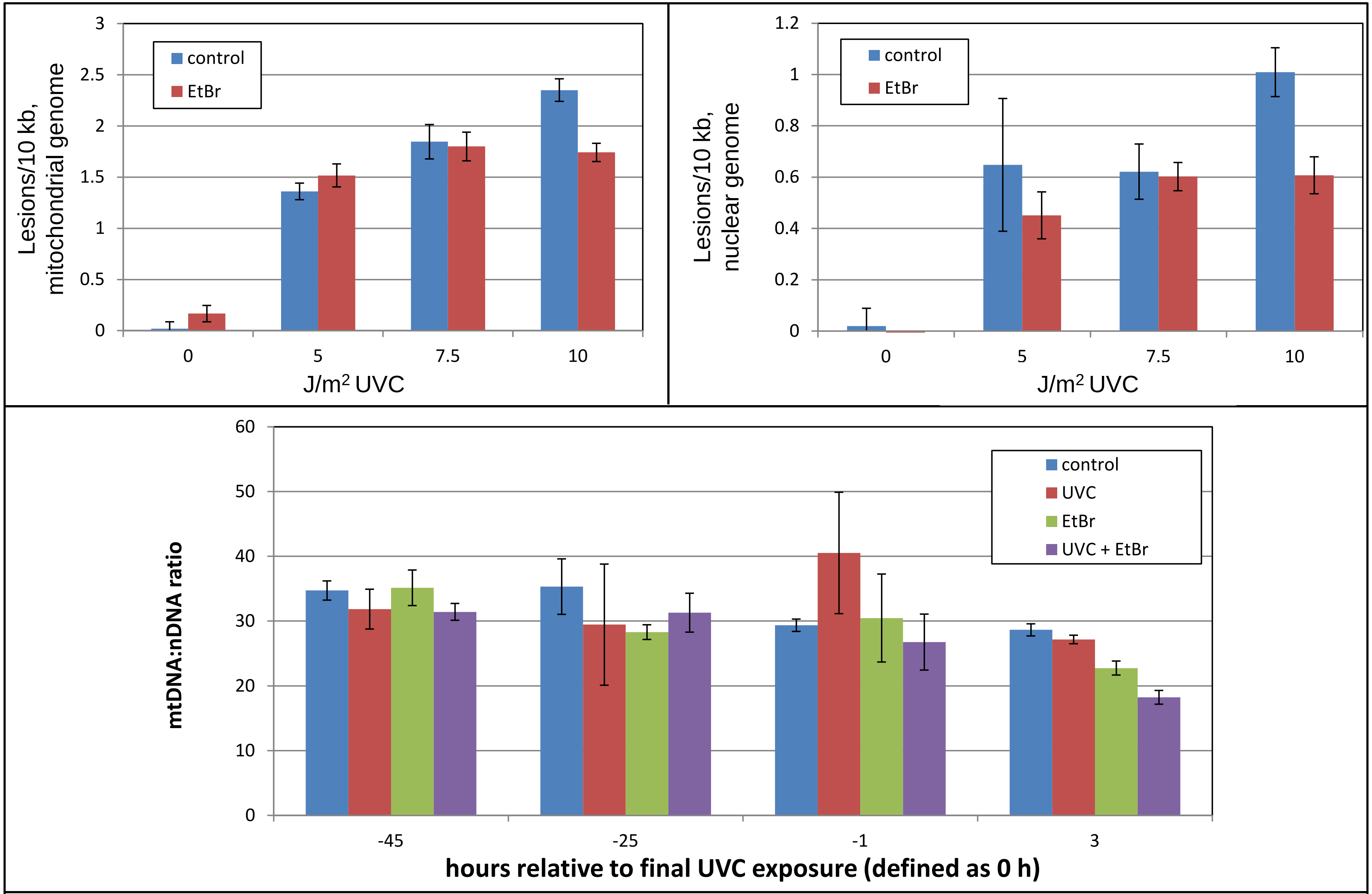

Additional Figure 2. Exposure to EtBr (5 µg/mL) did not result in measureable DNA damage (measured 1 h after the third UVC exposure) in either genome, nor did it exacerbate UVC-induced DNA damage in either genome ($p > 0.05$ for main effect of EtBr and interaction term, 2 factor ANOVA). The exposures did not result in a marked change in mtDNA:nDNA ratio (bottom panel); time ($p = 0.50$), treatment ($p = 0.91$) and the interaction term ($p = 0.89$) were all statistically insignificant. Therefore, we could not make direct within-time comparisons by FPLSD. n = 4-8 per bar.

Test Description

Selected Test : 3way ANOVA
p-value computation: Asymptotic
Multiple Testing Correction: Benjamini-Hochberg

Result Summary

	P all	P < 0.05	P < 0.01	P < 0.0010
Corrected p-value(time)	22625	14970	12542	10095
Corrected p-value(time-EtBr)	22625	211	12	3
Corrected p-value(time-EtBr-UV)	22625	0	0	0
Corrected p-value(time-UV)	22625	23	11	6
Corrected p-value(EtBr)	22625	6929	4095	1447
Corrected p-value(EtBr-UV)	22625	0	0	0
Corrected p-value(UV)	22625	140	77	36
Expected by chance		748	125	10

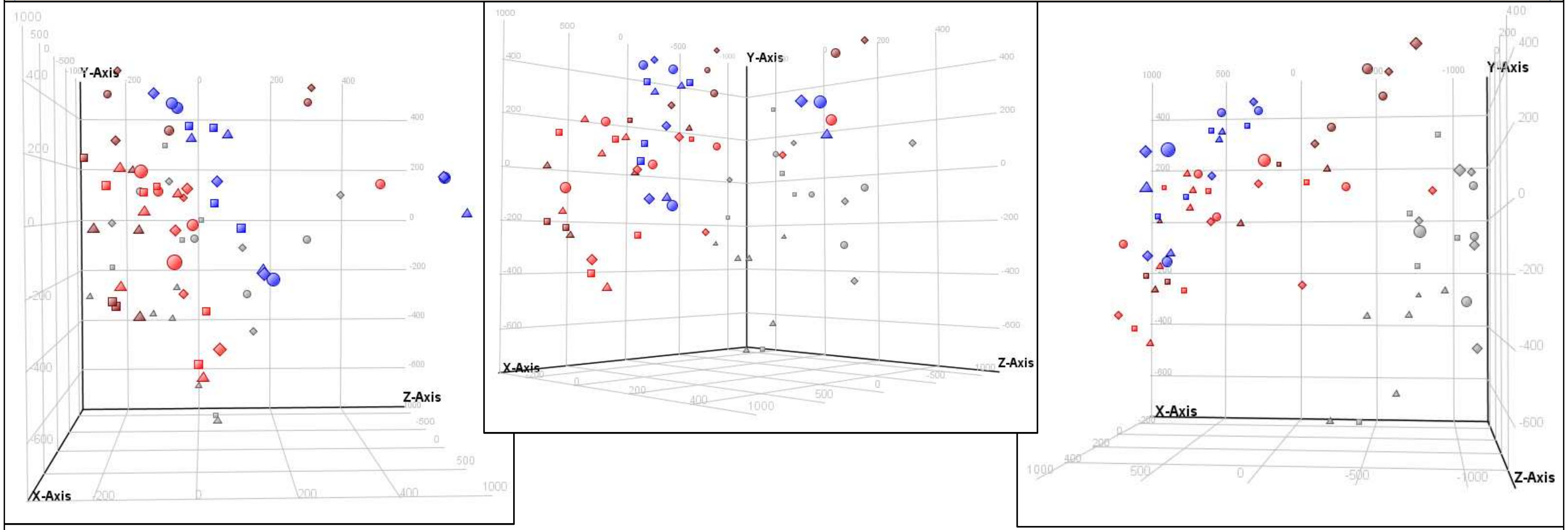

Additional Figure 3. Time and EtBr were the major drivers of differential gene expression by ANOVA and PCA. Even at the least stringent p-value, where 748 differentially expressed genes are expected to be identified by chance, no genes were differentially regulated in response to UVC in a way that was modulated by EtBr or EtBr and time. In the PCA (three views of same plot), the x-axis (component 1) explains 52% of variability, the y-axis 21%, and the z-axis 14%. Blue indicates -45 h, red -25 h, maroon -1 h, and grey 3 h. Diamonds indicate control samples, circles UVC, squares EtBr, and triangles UVC + EtBr. Analyses performed with GeneSpring.

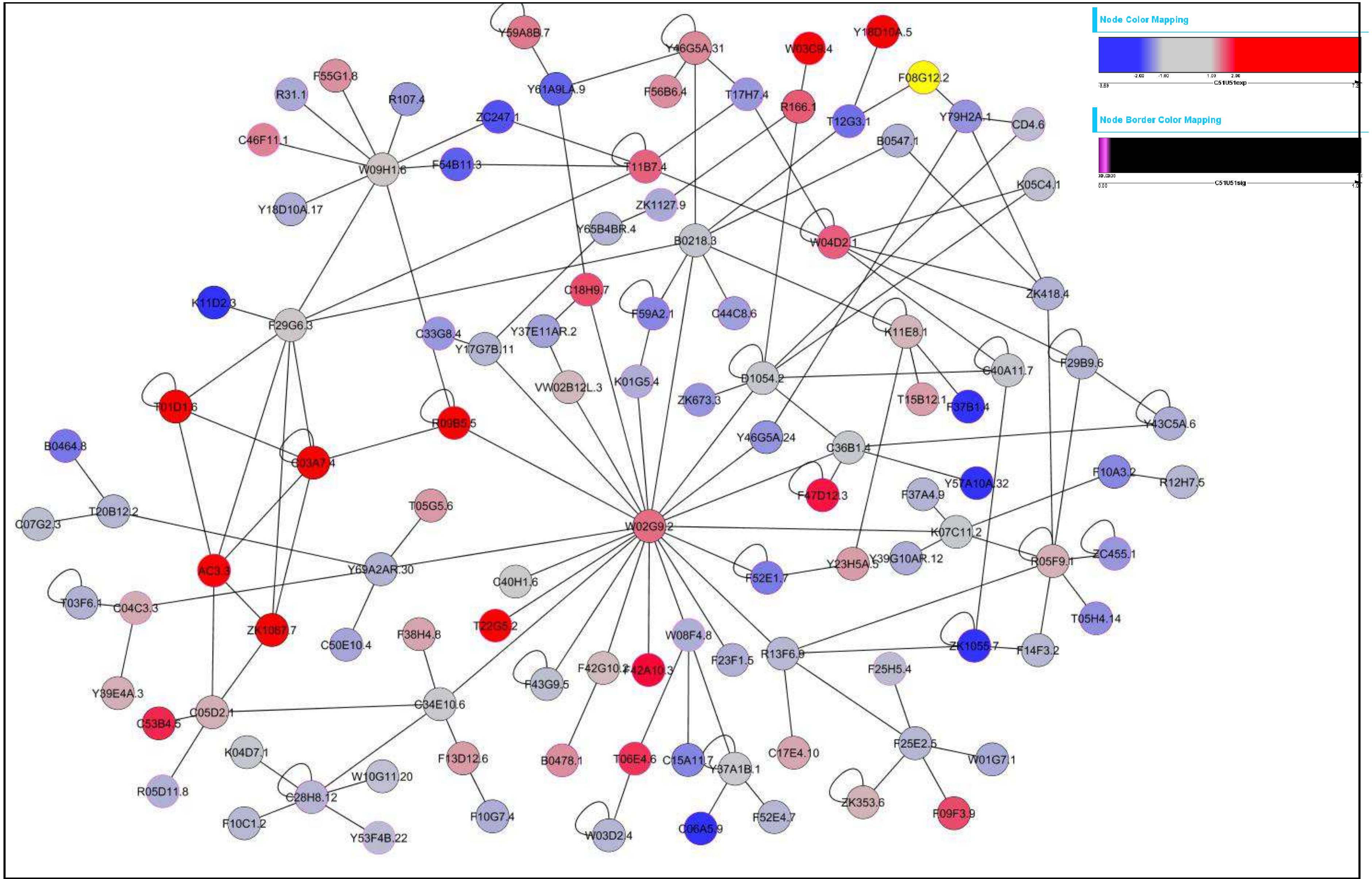

Additional Figure 4. At 3 h, the combination treatment led to additional effects compared to EtBr alone. Development is the most-altered gene ontology; differences were also observed in transcription, protein catabolism, and organellar organization. Blue indicates higher expression in the combination than in EtBr alone.

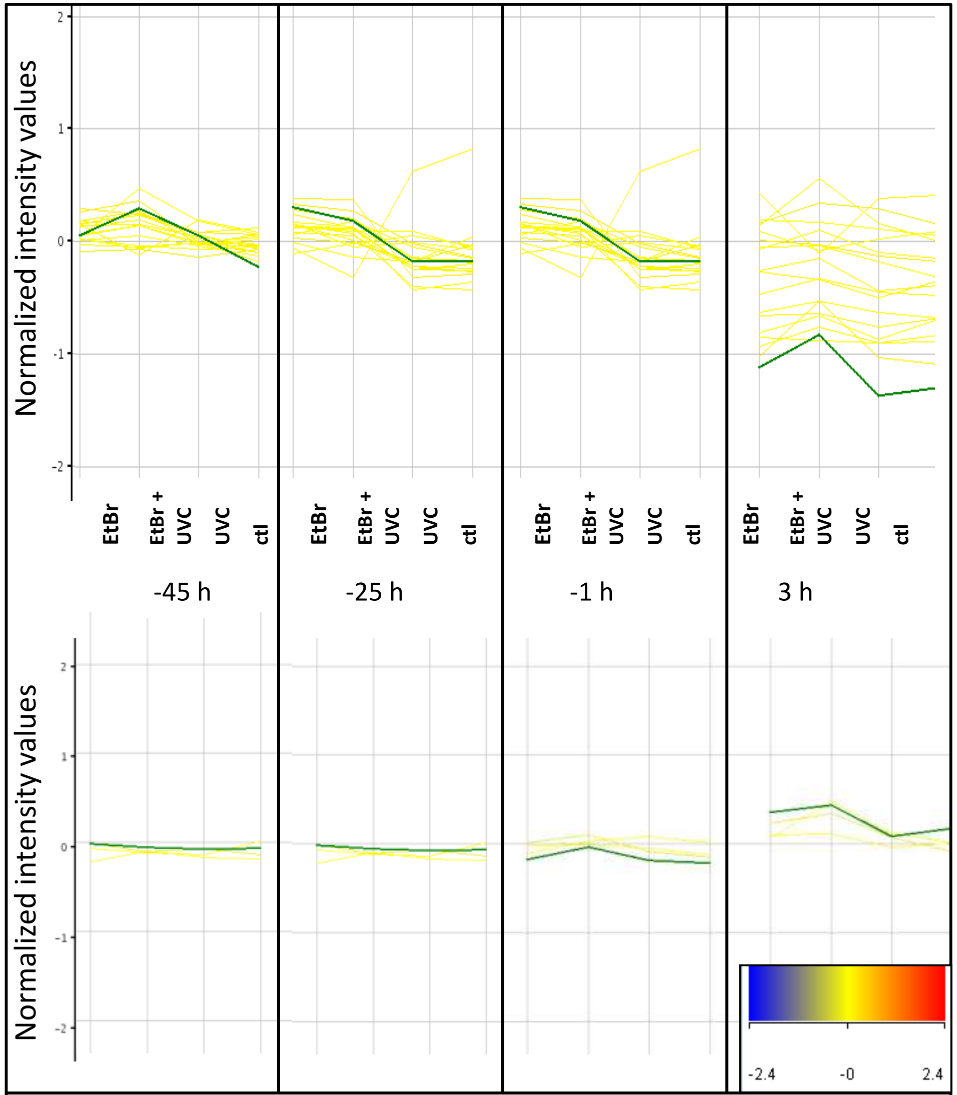

Additional Figure 5. Some autophagy genes were induced by EtBr and UVC at later timepoints (top panel; *lgg-2* highlighted). No changes were observed in fusion or fission genes (bottom panel; *eat-3* highlighted). Red-blue scale coloration is based on comparison to mRNA levels in control samples at -45 h. Normalized intensity values are on a binary log scale (i.e. “1” indicates a 2-fold change, “2” a 4-fold change, etc.). n = 4-6.

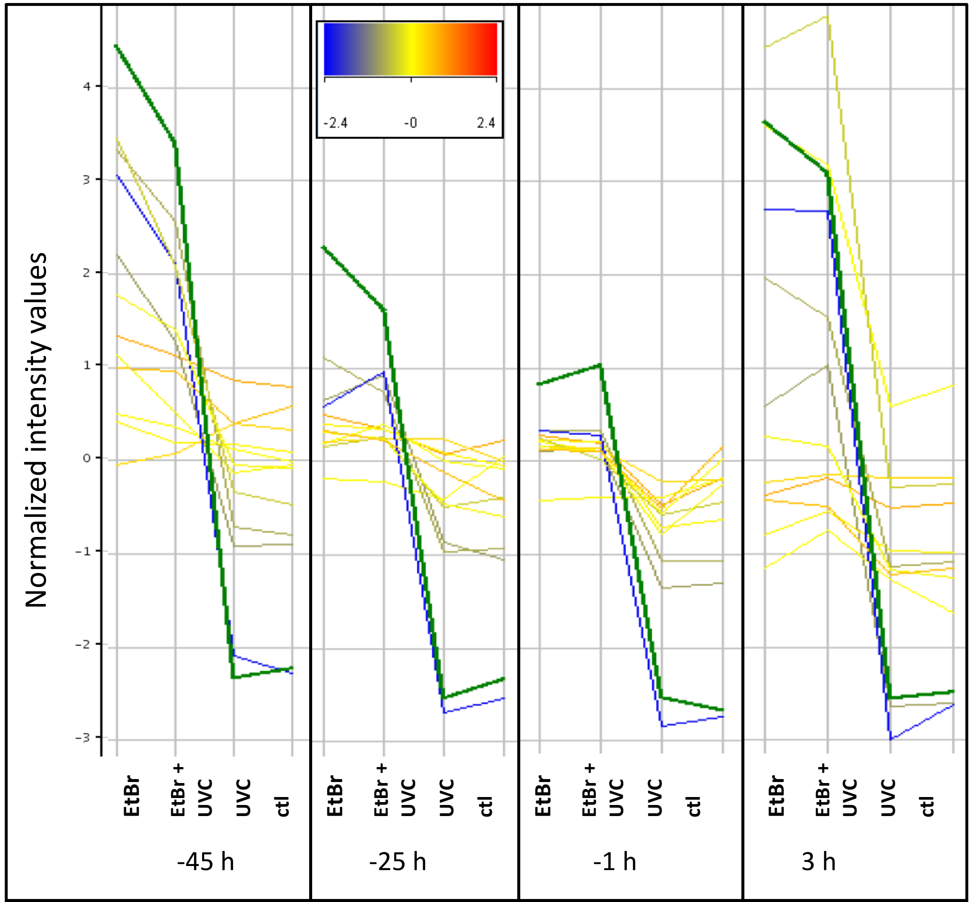

Additional Figure 6. Many cytochrome P450 genes were upregulated by exposure to ethidium bromide; shown are genes upregulated by EtBr and fitting the GO term “monoxygenase activity.” The genes shown are *cyp-35B3* (highlighted in green), *cyp-13A7*, *cyp-35A5*, *cyp-35B1*, *cyp-33C3*, *cyp-33C6*, *cyp-33C7*, *cyp-33D3*, *cyp-35B2*, *cyp-35A1*, *cyp-33C5*, and *cyp-33C4*. Red-blue scale coloration is based on comparison to mRNA levels in control samples at -45 h. Normalized intensity values are on a binary log scale (i.e. “1” indicates a 2-fold change, “2” a 4-fold change, etc.). n = 4-6.

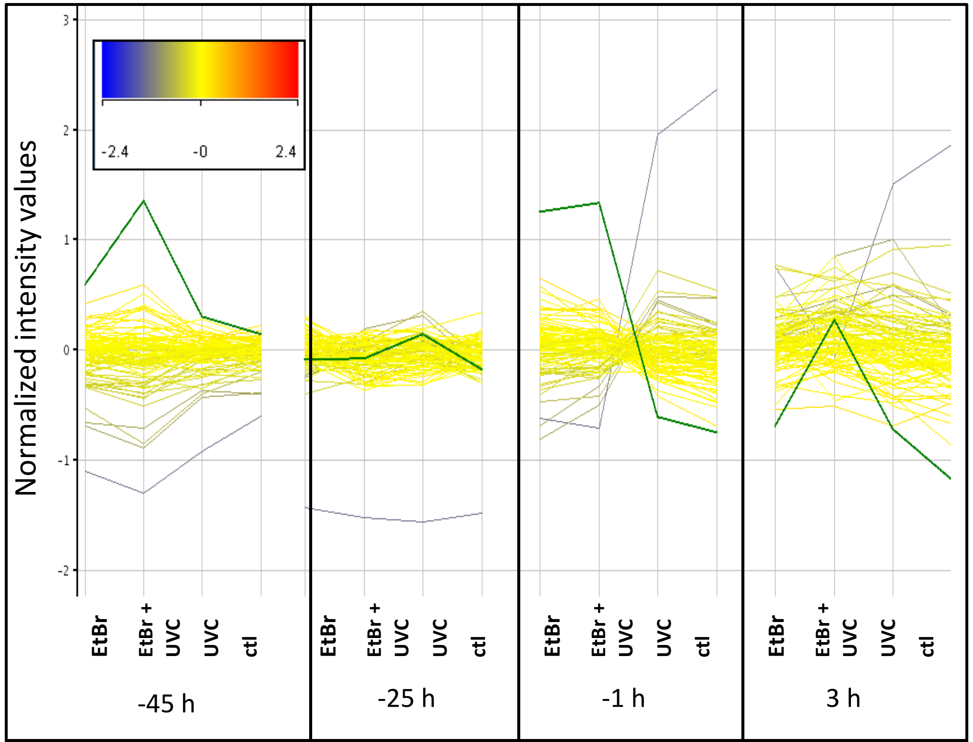

Additional Figure 7. There is little change in expression of known DNA repair genes (from Boyd et al., 2010) either with time or treatment, with the exception of *pme-4* (highlighted). Red-blue scale coloration is based on comparison to mRNA levels in control samples at -45 h. Normalized intensity values are on a binary log scale (i.e. “1” indicates a 2-fold change, “2” a 4-fold change, etc.). n = 4-6.

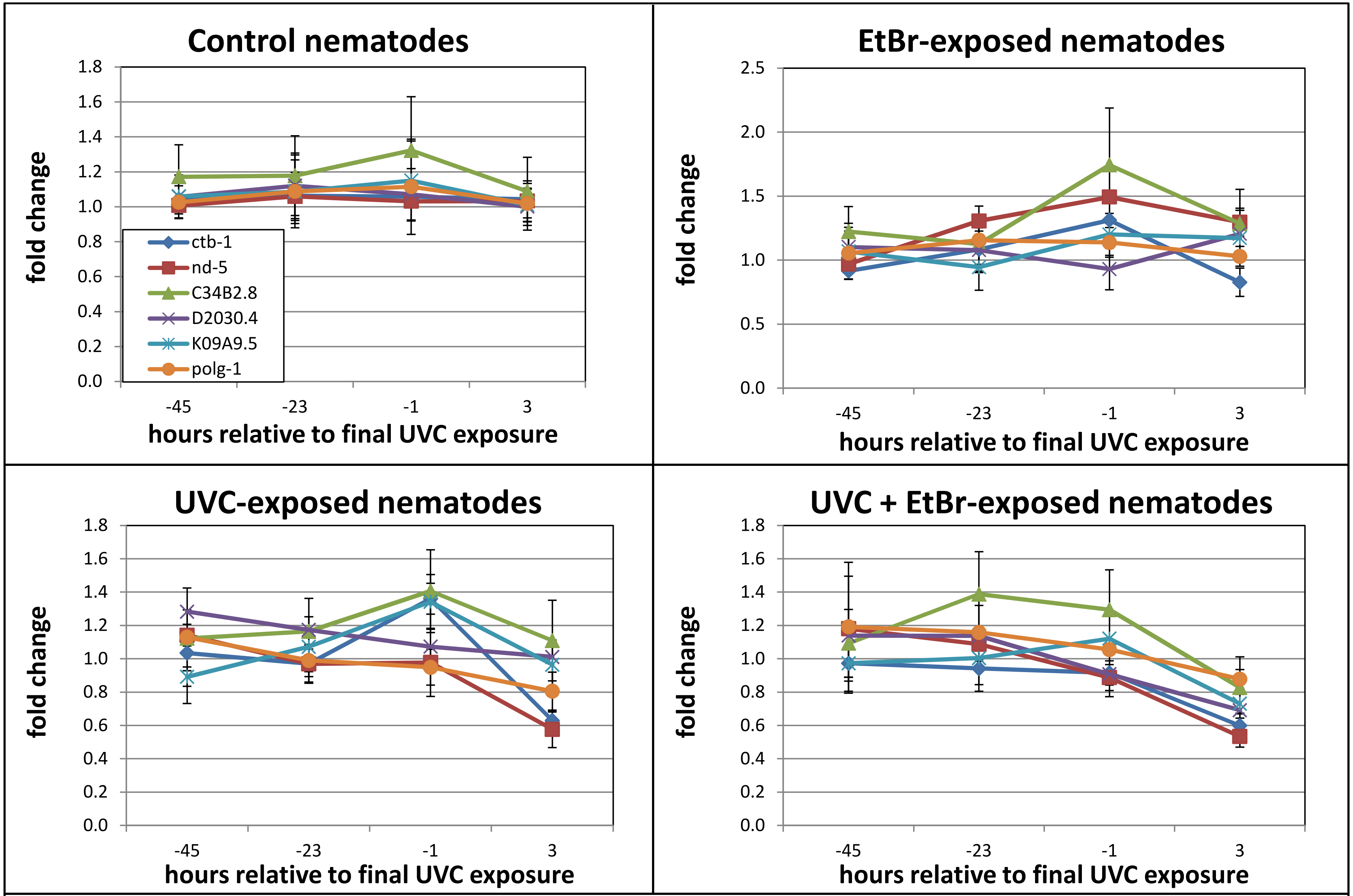

Additional Figure 8. Effect of exposure to UVC, EtBr or both on mRNA levels for mtDNA-encoded (*ctb-1*, *nd-5*) and nDNA-encoded (C34B2.8, D2030.4, K09A9.5, *polg-1*) mitochondrial proteins. The legend is the same for all graphs. $p < 0.05$ for main effects of time, treatment, and genome, and genome x time interactions. Fold change is relative to the mRNA of the same gene at the same time without UVC exposure. n = 5-7 (samples derived from microarray experiment exposures, including additional samples not used for microarray).

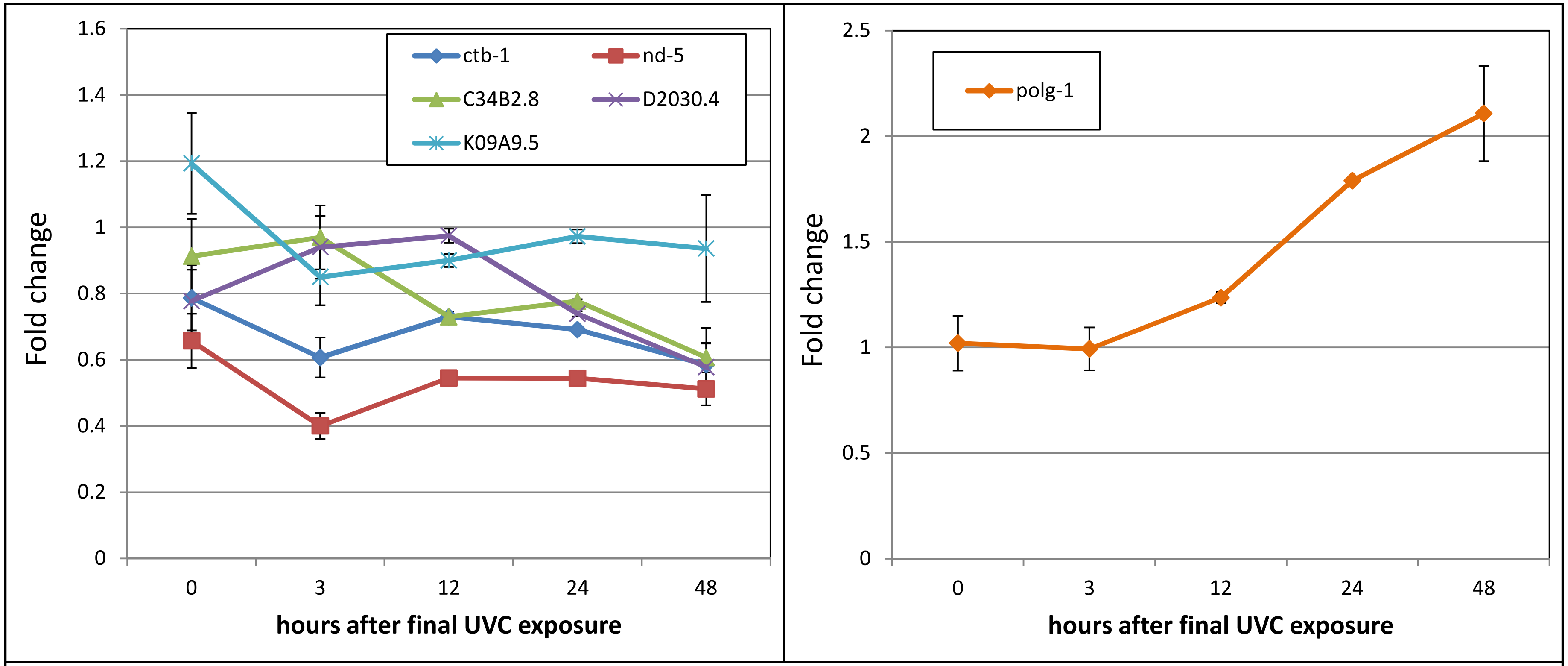

Additional Figure 9. Effects of exposure to UVC in the PE255 strain. *polg-1* is graphed separately for consistency with Fig. 6. *ctb-1* and *nd-5* are mtDNA-encoded, and C34B2.8, D2030.4, K09A9.5, *polg-1* are nDNA-encoded, mitochondrial proteins. The effect of UVC and gene ($p = 0.016$ and $p < 0.0001$ respectively) were significant, as was the interaction of UVC with gene ($p = 0.003$). No other main or interactive effects were significant ($p > 0.05$, 3 factor ANOVA). Comparisons at specific times could not be made due to the lack of significant interactions involving time. Gene-by-gene comparisons (across time) by FPLSD indicated that *polg-1* behaved differently than all other genes ($p \leq 0.003$), and *nd-5* was distinct from K09A9.5 ($p = 0.02$). Fold change is relative to the mRNA of the same gene at the same time without UVC exposure. n = 3-6.
